# Exploring the spatiotemporal structure and driving mechanism of digital village construction in China based on social network analysis and Geodetector

**DOI:** 10.1371/journal.pone.0310846

**Published:** 2024-11-15

**Authors:** Liping Zhang, Xiaoliang Zhou

**Affiliations:** 1 School of Economics and Trade, Fujian Jiangxia University, Fuzhou, China; 2 Fujian Southeast Digital Economy Research Institute, Fuzhou, China; 3 School of Economics and Management, Fuzhou University, Fuzhou, China; Tallinn University of Technology School of Engineering: Tallinna Tehnikaulikool Inseneriteaduskond, ESTONIA

## Abstract

Clarifying the spatiotemporal structure and driving mechanism of China’s digital village construction (DVC) is imperative for ameliorating regional disparities and fostering the holistic progression of DVC in China. This study assesses the index of DVC in 30 Chinese provinces from 2011 to 2021 using the Intuitionistic Fuzzy Analytic Hierarchy Process (IFAHP) and dynamic GRA. It analyzes the spatiotemporal structure of DVC with kernel density, trend surface, and social network analysis techniques. Additionally, it employs Geodetector to elucidate the driving mechanism behind spatial differentiation in China’s digital village development network. The results indicate that: (1) Although the index of DVC in China from 2011 to 2021 has shown progressive enhancement, the average DVC index for all regions throughout the years surveyed stands at 0.457, which means that the DVC in China is still at an early stage. (2) The overall network structure analysis suggests that the number of ties in China’s DVC spatial correlation network grew slowly but still falls significantly short of the ideal number. Additionally, there is an increase in the network density of China’s DVC over the years, providing strong evidence of spatial spillover effects within the network. (3) The block roles of the central and western regions are main inflow and bidirectional spillover while the block roles of the eastern region are agent and main outflow. (4) The main driving factors of DVC in China are investment in information infrastructure and fiscal expenditure on education. Bivariate enhancement effect and nonlinear enhancement were found to exist in all interactions of indicators. These findings offer theoretical insights and practical directives for improving DVC in China and its synergistic effects.

## Introduction

Over an extended period, the constraints posed by China’s urban-rural dual system have resulted in disparities in development between urban and rural areas [1]. The advancement of digital technology presents an opportunity to diminish the urban-rural divide and achieve balanced development between urban and rural areas. In 2018, the Chinese central government for the first time proposed to expedite the implementation of a rural digitalization strategy. Subsequently, a series of policy measures were introduced, including the issuance of the "Outline of the Digital Rural Development Strategy" in May 2019, which advocated for the "implementation of national digital rural pilot projects." Additionally, in July 2021, the release of the "Digital Rural Construction Guidelines 1.0" took place, followed by the unveiling of the "Digital Rural Development Action Plan (2022–2025)" in January 2022. These initiatives aim to narrow the urban-rural digital divide, leverage digital technology to address agricultural and rural issues, and comprehensively enhance the high-quality development of China’s rural economy and society.

Digital village construction (DVC) is conducive to unleashing the potential of information technology in promoting agricultural upgrading and driving profound changes in agricultural production and rural social life. For instance, through the digitization of agricultural machinery, the automation and intelligence levels of traditional agricultural machinery can be enhanced, leading to increased efficiency in agricultural production. Establishing an automatic plant cultivation system through digital technologies can dynamically adjust crop growth environments, conserve resources such as fertilizers and water, and reduce environmental pollution. Online sales platforms can broaden the channels for agricultural product distribution and boost income for rural residents. The "Internet Plus Education" mode can provide rural students with access to high-quality online educational resources, thereby enhancing educational equity and reducing limitations in accessing educational information for rural residents. Furthermore, DVC can increase non-agricultural employment opportunities for rural residents through online information channels, thereby enhancing the flexibility of rural labor force employment [2].

After years of sustained investment, China’s rural digital infrastructure has made significant progress, with digital technology deeply integrated into various aspects of rural residents’ daily production and life, becoming a crucial driving force for the construction and development of the "new countryside". According to data released by the Ministry of Agriculture and Rural Affairs in November 2020, in 2019, the amount of agricultural products sold through the Internet by rural residents in China reached 10% of the total national sales of agricultural products, and the coverage rate of e-commerce service outlets in villages nationwide had reached 74% [3]. According to the latest data from the National Bureau of Statistics, as of 2022, the average Internet coverage rate in rural areas of China has exceeded 70%, and the average number of smartphones per hundred households in rural areas has exceeded 260. Despite some progress in China’s digital rural development, the vast expanse and uneven economic and social development levels across rural areas lead to disparities in the level of digital rural development. What is the present condition of digital village development in China? How is the temporal and spatial structure of digital village development in China? What are the driving mechanisms behind digital village development? Answering these questions could be beneficial for a comprehensive understanding of the current status of DVC in China, as well as for clarifying the temporal and spatial structures of DVC. This carries certain significance for local governments to design tailored digital rural development strategies and for the central government to develop policies that promote interregional coordination in the advancement of digital village development.

Currently, research on the connotation of digital village is still in the exploratory stage [4]. Sutriadi conceptualized the digital village as an innovative approach to attain sustainable development in rural areas by applying digital technologies for the efficient development of different parts of the village [5]. Some other scholars argue that the essence of DVC lies in the modernization of agriculture and rural regions, characterized by the advancement of contemporary information technology. Therefore, enhancing overall digital infrastructure, and improving villagers’ information literacy are essential to bolster the intrinsic dynamics of rural development [6]. DVC facilitates the integration of digital technology into the production and daily life of rural residents, contributing to the realization of agricultural modernization [7]. DVC is an essential pathway for achieving rural revitalization in the context of the digital economy, through fostering integrated urban-rural development and digitizing agricultural production [8]. Some other scholars have also paid attention to the impact of DVC on rural development. For example, Zhao et al. studied the influence of digital rural development on farmers’ entrepreneurship and found that digital village development significantly increases the probability of villagers’ entrepreneurship, and the digitization of rural infrastructure has a positive impact on villagers’ entrepreneurial decision-making [9]. Goyal’s research on rural residents in India found that increasing rural digital infrastructure effectively improves villagers’ income [10]. Zhou analyzed provincial panel data from 2011 to 2021 in China to examine the impact of digital rural development on the resilience of the agricultural economy and found that DVC can enhance the resilience of the agricultural economy by increasing agricultural labor productivity [11]. Another topic that scholars paid much attention to was the measurement of the level of digital rural development. Fang constructed an evaluation index system for digital rural development from the perspective of rural development [12]. This system comprises seven dimensions, namely, digital agriculture, smart farmers, digital economy, online governance, digital environment, digital healthcare and welfare, and infrastructure [12]. Shen et al. developed an index system for digital villages considering factors such as the utilization of information equipment, the acquisition and utilization of digital information, and the provision of public information services, among others [13]. Cui and Feng constructed an assessment index system for DVC consisting of four dimensions: digital services, digital investment, digital benefits, and digital environment [14]. Zhang et al. devised a DVC evaluation index system by considering digital environment, digital infrastructure, digital government, information environment, etc [15].

It can be found that existing literature on the measurement indicators for China’s digital village development primarily focuses on information access and the digital environment, lacking attention to dimensions such as rural digital production and living. Given that rural residents are the primary participants in digital rural development, we believe that the evaluation of DVC should take into account dimensions directly related to rural residents’ digitized agricultural production, digitized agricultural product sales, digitized lifestyle. Additionally, we suppose that the potential for rural digitalization development is also an essential dimension for measuring digital rural development. For instance, night-time light intensity of a region observed by satellites was considered to authentically reflect human activities, as well as the socio-economic development status and development potential of the region [16,17]. Therefore, indicators such as night-time light intensity and educational level of rural residents could serve as indicators, which could effectively assess the potential for rural digitalization development of regions. Furthermore, previous studies predominantly investigated the theory and practices of digital village development from economic and management perspectives. However, there is a lack of investigation into the network patterns of regions’ digital rural development from a dynamic temporal and spatial perspective.

The current study intends to construct a comprehensive evaluation index system for digital rural construction in China. IFAHP (Intuitionistic Fuzzy Analytic Hierarchy Process) was employed to determine the weights of indicators and the dynamic GRA method was utilized to assess the values of digital village development of 30 provinces of China from 2011 to 2021. We also analyze the regional structural characteristics of China’s digital rural development from the perspective of spatiotemporal correlation. For example, we used kernel density estimation to illustrate the overall distribution of DVC in the target areas and employed Ucinet 6 to analyze the regional spatial network structure of DVC in China. Geodetector was also used to explore the influencing mechanism of spatial stratified heterogeneity of DVC in China. By doing so, we hope to enrich the theories related to DVC and to offer inspiration for governments to formulate regional coordination policies that elevate the level of DVC in China and reduce regional disparities.

## Methods

### IFAHP

The evaluation index system for DVC of China is a complex system covering aspects such as agriculture, finance, trade, and education. Due to the complexity and diversity of its evaluation elements, experts may not possess all the necessary knowledge required to evaluate all indicators. Consequently, they may experience some hesitation during the evaluation process. IFAHP (intuitionistic fuzzy analytic hierarchy process) is an extension of the FAHP (fuzzy analytic hierarchy process), which can better handle uncertainty during the evaluation process [18]. Therefore, this study adopts IFAHP to calculate the weights of indicators for the level of DVC in China.

Suppose set X is an intuitionistic fuzzy set, which is a non-empty set. *t*_*A*_(*x*) and *f*_*A*_(*x*) respectively represent the membership degree and non-membership degree of an element x in the subset A of set X, satisfying: $A=\{\langle x,t_{A}(x),f_{A}(x)\rangle |x\in X\},0⩽t_{A}(x)⩽1,0⩽f_{A}(x)⩽1,\;\mathrm{and}\;t_{A}+f_{A}+\pi_{A}=1$, in which *x*∈ X, *π*_*A*_ represents the hesitation degree. The main calculation process is as follows:

Step 1: Establish the intuitionistic fuzzy judgment matrix: $R={\left(r_{gh}\right)}_{n\times n}={\left(t_{gh},f_{gh}\right)}_{n\times n}$, Where *g* and *h* respectively represent the rows and columns of the matrix, and *n* represents the number of indicators at the criterion layer.

Step 2: Test the consistency of the matrix, judging by the formula:

$$d(R,\bar{R})=\frac{1}{2(n-1)(n-1)}\sum_{g=1}^{n}\sum_{h=1}^{n}\left(|{\bar{t}}_{gh}-t_{gh}|+\left|{\bar{f}}_{gh}-f_{gh}\right|+\left|{\bar{\pi }}_{gh}-\pi_{gh}\right|\right)$$ (1)

Here, $R={\left(r_{gh}\right)}_{n\times n}$ is the intuitionistic fuzzy judgment matrix, $\bar{R}={\left(\bar{r_{gh}}\right)}_{n\times n}$ is the judgment matrix, ${\bar{t}}_{gh}$, ${\bar{f}}_{gh}$ and ${\bar{\pi }}_{gh}$ represent the membership degree, non-membership degree, and hesitation degree. ${\bar{t}}_{gh}$ and ${\bar{f}}_{gh}$ can be obtained by:

(1) When *h*>*g*+1,

$${\bar{t}}_{gh}=\frac{\sqrt[{h-g-1}]{\prod_{v=g+1}^{h-1}t_{gv}\times t_{vh}}}{\sqrt[{h-g-1}]{\prod_{v=g+1}^{h-1}t_{gv}\times t_{vh}}+\sqrt[{h-g-1}]{\prod_{v=g+1}^{h-1}\left(1-t_{gv}\right)\times (1-t_{vh})}}$$ (2)

$${\bar{f}}_{gh}=\frac{\sqrt[{h-g-1}]{\prod_{v=g+1}^{h-1}f_{gv}\times f_{vh}}}{\sqrt[{h-g-1}]{\prod_{v=g+1}^{h-1}f_{gv}\times f_{vh}}+\sqrt[{h-g-1}]{\prod_{v=g+1}^{h-1}\left(1-f_{gv}\right)\times (1-f_{vh})}}$$

(2) When *h* = *g*+1 or *h* = *g*, set ${\bar{r}}_{gh}=r_{gh}$.
(3) When *h*≤*g*+1, set ${\bar{r}}_{gh}=({\bar{f}}_{hg},{\bar{t}}_{hg})$.

If $d(R,\bar{R})<\tau$, it indicates that the matrix passes the consistency test, where *τ* is the threshold for the consistency test, typically set at 0.01. If the consistency test is not passed, then the matrix should be adjusted according to formula (3) [19].


$${\tilde{t}}_{gh}=\frac{{\left(t_{gh}\right)}^{1-\sigma }{\left({\bar{t}}_{gh}\right)}^{\sigma }}{{\left(t_{gh}\right)}^{1-\sigma }{\left({\bar{t}}_{gh}\right)}^{\sigma }+{\left(1-t_{gh}\right)}^{1-\sigma }{\left(1-{\bar{t}}_{gh}\right)}^{\sigma }}$$ (3)

$${\tilde{f}}_{gh}=\frac{{\left(f_{gh}\right)}^{1-\sigma }{\left({\bar{f}}_{gh}\right)}^{\sigma }}{{\left(f_{gh}\right)}^{1-\sigma }{\left({\bar{f}}_{gh}\right)}^{\sigma }+{\left(1-f_{gh}\right)}^{1-\sigma }{\left(1-{\bar{f}}_{gh}\right)}^{\sigma }}$$

Here,σ∈[0,1], and the value of σ could be adjusted iteratively until the matrix passes the consistency test.

Step 3: Compute the weight vector of each intuitionistic fuzzy judgment matrix. Following the calculation process used by scholars like Xu and Liao [20], we obtained the weight vector of each matrix by the formula (4):

$$\omega_{g}=(t_{g},f_{g})=(\frac{\sum_{h=1}^{n}t_{gh}}{\sum_{g=1}^{n}\sum_{h=1}^{n}\left(1-f_{gh}\right)},1-\frac{\sum_{h=1}^{n}\left(1-t_{gh}\right)}{\sum_{g=1}^{n}\sum_{h=1}^{n}f_{gh}}),g=1,2,\ldots ,n.$$ (4)

Then compute the weight of each indicator belonging to the same upper-level indicators by formula (5):

$$G_{g}=\frac{1-f_{g}}{1+\pi_{g}},g=1,2,\ldots n$$ (5)

where *π*_*p*_ = 1−*t*_*p*_−*f*_*p*_. Finally, normalize the weight of each indicator according to formula (6):

$$\lambda =\frac{G_{g}}{\sum_{g=1}^{n}G_{g}}$$ (6)

### Dynamic GRA

GRA (Grey Relational Analysis) was initially proposed by Deng in 1982 [21], with a focus on resolving issues involving unknown factors. GRA concentrates on investigating uncertain systems characterized by "partially known and partially unknown information" [21]. It describes the operational patterns of systems by extracting usable information from the already known data. GRA is applicable for studying grey systems with multiple levels and complex mechanisms [21].

Given that the data utilized in this study are dynamic panel data, it is necessary to consider the dynamic changes in China’s digital rural development over time based on the same evaluation criteria, so as to effectively compare and analyze the statistical results of DVC from different years. Referring to the methods of panel data processing proposed by Shi et al. [22], this paper employs the dynamic GRA to calculate the digital village development levels of various provinces of China for each year. The calculation method is as follows:

Step 1: To address the incomparability resulting from diverse dimensions, it is necessary to standardize the data of all indicators. The standardization formula for positive indicators is $b_{ij}^{k}=\frac{b_{ij}^{k}-b_{i}^{-}}{b_{i}^{+}-b_{i}^{-}}$. For negative indicators, the formula is $b_{ij}^{k}=1-\frac{b_{ij}^{k}-b_{i}^{-}}{b_{i}^{+}-b_{i}^{-}}$. $b_{ij}^{k}$ refers to the value of the *i*-th indicator for the *j*-th province//municipality in the *k*-th year. $b_{i}^{-}=\underset{1\leq k⩽T}{\min}(b_{i}^{k})$ represents the minimum score value for the *i*-th indicator across all *T* years. $b_{i}^{+}=\underset{1\leq k⩽T}{\max}(b_{i}^{k})$ signifies the maximum score value for the *i*-th indicator across all *T* years of the panel data.

Step 2: Identify a reference sequence and compute the matrix of sequence differences. The reference sequence in this study is composed of the ideal evaluation matrices from the panel data, $\left(B^{+}=(b_{i}^{+})\right)$.

The sequence difference matrix Δ_*ij*_ is obtained by subtracting the optimal values of the corresponding indicators in all years from $b_{ij}^{k}$ in the panel data. The formula for calculating the difference sequence matrix is: Δ_*ij*_ = | $b_{ij}^{k}$-$b_{i}^{+}$|.

Step 3: Determine the grey relational coefficient matrix and calculate the grey relational coefficients between each evaluation object and the positive ideal solution. The formula is:

$$\xi_{ij}=\frac{\underset{i}{\min}\underset{j}{\min}\Delta_{ij}+p\underset{i}{\max}\underset{j}{\max}\Delta_{ij}}{\Delta_{ij}+p\underset{i}{\max}\underset{j}{\max}\Delta_{ij}}$$ (7)

Where *p* is the resolution coefficient, *p*∈[0,1], typically taken as 0.5.

Step 4: Determine the comprehensive evaluation score for digital rural construction in each province for every year by conducting the multiplication of the grey relational coefficient matrix with the weight matrix derived from the panel data’s indicators. The calculation formula is:

$$r_{j}^{k}=\sum_{i=1}^{n}\lambda_{i}\times \xi_{ij}^{k}$$ (8)

Here, $r_{j}^{k}$ represents the comprehensive assessment value of DVC for *j*-th province//municipality in the *k*-th year, $r_{j}^{k}\in [0,1]$. The closer $r_{j}^{k}$ is to 1, the higher the level of digital rural construction of the *j*-th province in the *k*-th year. *n* means the number of indicators. *λ*_*i*_ signifies the weight of the *i*-th indicator. $\xi_{ij}^{k}$ indicates the grey correlation coefficient between the *i*-th indicator for the *j*-th province//municipality in the *k*-th year and its corresponding positive ideal solution.

### Social network analysis

Although traditional spatial correlation analysis methods can identify the spatial relationships between regions, they cannot reveal the underlying structural features of spatial correlations [23]. The social network analysis method is a quantitative analysis method based on mathematical methods, graph theory, and others. Social network analysis method quantitatively analyzes various relationships within a network to examine the overall characteristics of the network and analyze the positions of individual subjects within the network [24]. It is commonly used to investigate the structural and attribute characteristics of a network with groups of social actors. Characterization of spatial correlation network features mainly involves methods such as overall network structure characteristics, individual network structure characteristics and block model analysis [25]. Due to the varying levels of political, economic, cultural, and technological exchanges between different regions in China, there inevitably exist spatial network relationships in the levels of DVC among various regions in China. Based on the social network analysis method, this paper explores the regional social network structure and characteristics of the construction of digital villages in China.

The gravity model is an important quantitative model in geography for revealing spatial interactions between cities [26]. According to the gravity model, the levels of the spatial interactions between subjects are directly proportional to the mass between the two subjects and inversely proportional to the distance [26]. This study draws upon an improved gravity model to describe the spatial correlation of DVC between regions in China:

$$W_{ij}=\frac{\sqrt{G_{i}}\sqrt{G_{j}}}{{\left(D_{ij}\right)}^{2}}$$ (9)

Here, *W*_*ij*_ represents the gravity values of DVC of provinces/ municipalities of China, namely the spatial correlation strength. *D*_*ij*_ is the distance between region *i* and region *j*, *G*_*i*_ signifies the DVC index of region *i*, and *G*_*i*_ indicates the DVC index of region *j*.

After calculating the gravity matrix according to Eq (9), we developed the spatial binary matrix for DVC of China using a binary method. We took the average value of the data in each row of the matrix as the threshold value for that row. If the gravity is higher than the threshold value of that row, the value is set to 1, indicating the existence of a correlation in digital rural construction between region *i* and region *j*. Otherwise, it is set to 0, indicating no correlation in digital rural construction between region *i* and region *j*.

### Geodetector model

Geodetector is a spatial variance analysis approach, assessing the spatial coherence between independent and dependent variables. Essentially, if one spatial area’s independent variable impacts another, the dependent variable’s spatial distribution in the latter area will resemble that of the independent variable [27]. Geodetector model comprises four sub-detectors: factor detection, risk detection, interaction detection, and ecological detection. This study mainly utilized factor detection and interaction detection. Factor detection primarily utilizes the q-statistic to examine the extent to which explanatory variables can explain the spatial heterogeneity of the response variable within the observed area. The formula is as follows:

$$q=1-\frac{\sum_{x=1}^{M}N_{x}\sigma_{x}^{2}}{N\sigma^{2}}$$ (10)

In Formula (10), *x* = 1,2,3…*M*, and represents the number of sub-regions obtained by stratifying the study area, *N*_*x*_ and *N* are the number of units in layer x and the number of units in the entire region respectively. $\sigma_{x}^{2}$ and σ^2^ respectively represent the variance of layer x and the variance of the values of the entire region. "q ≥ 0.3" indicates that the explanatory variable has relatively strong explanatory power.

The value of *q* signifies the explanatory power of the independent variable on the dependent variable, ranging from [0, 1]. If an independent variable is completely unrelated to the dependent variable, *q* = 0; if the explanatory variable has complete explanatory power over the dependent variable, then *q* = 1. If q ≥ 0.3, it suggests that the explanatory variable has relatively strong explanatory power [28].

Interaction detection can identify the interactive effects of different spatial explanatory variables on spatial dependent variables. They can also assess whether the combined interaction of two explanatory variables strengthens or weakens their explanatory power on the dependent variable. For example, the interaction between two explanatory variables, A1 and A2, could be analyzed by comparing their individual values and the combined value (A1∩A2) to determine whether the interaction has a strengthening or weakening effect.

### Selection of evaluation factors and data sources

To construct a scientifically sound evaluation index system is essential for assessing the level of digital village development [29]. Based on the results of relevant research [30,31] and taking into account the current situation of digital rural development in China., this paper constructs a comprehensive evaluation index system for DVC in China. Rural residents play a crucial role as both participants and implementers of DVC [31]. Therefore, the assessment of digital rural construction should take into consideration the actual impact of digital technologies on the impact of digital technology on agricultural production, agricultural product sales, and residents’ daily lives. Drawing upon the accessibility of data and scientific principles, the index system constructed in this paper is primarily designed from the perspective of rural residents’ productive and daily life practices. In specific terms, the primary indicators comprise rural digital infrastructure, rural production digitization, rural distribution digitalization, rural marketing digitalization, rural life service digitalization, and Rural digitalization development potential. The secondary indicators encompass 22 factors (Table 1). Digital infrastructure is the prerequisite of digital rural development, and consequently, it should be a crucial indicator for measuring the level of digital rural construction. The secondary indicators of rural digital infrastructure include rural internet adoption rate, optical fiber network coverage rate, etc. Rural production digitalization is also an important primary indicator which includes agricultural production environment monitoring, electrification in agricultural production, and total power of agricultural machinery. The agricultural production environment monitoring is represented by the number of environmental and agricultural meteorological observation sites as a proxy indicator. Electrification in agricultural production is measured by the ratio of the added value of agriculture, forestry, animal husbandry, and fishery to the total rural electricity consumption. The secondary indicators of rural commodity circulation digitalization include rural postal and communication service level, rural consumer goods retail level, length of rural postal delivery routes, and length of rural postal delivery routes. Rural postal and communication service level is measured by the average population served per postal service outlet in rural areas. The secondary indicators of rural marketing digitalization mainly consist of e-commerce-related indicators such as the number of Taobao villages and rural e-commerce sales. Rural life service digitalization primarily assesses how digital tools improve the convenience of rural residents’ lives, while the potential for rural digitalization development is gauged by rural residents’ education level, technology access, rural financial investment, villagers’ net income, and night-time light intensity of rural areas. Night-time light intensity observed by satellites of a specific location can accurately reflect the social and economic development level of that area [32]. Therefore, this study incorporated night-time light intensity of rural areas into the assessment indicator system to evaluate the overall status of rural economic and social development, as well as their potential for digital development, of provinces in China.

**Table 1:** Evaluation index system for DVC in China.

Objective layer	Primary indicator and weight		Secondary indicator and weight	
	Primary indicator	Weight	Secondary indicator	Weight
Digital village development	Rural digital infrastructure (A1)	0.1596	Rural Internet adoption rate (B1)	0.2778
			Optical fiber network coverage rate (B2)	0.2419
			Number of smartphones per hundred households in rural areas (B3)	0.1966
			Number of computers per hundred households in rural areas (B4)	0.1607
			Rural cable television penetration rate (B5)	0.1230
	Rural production digitalization (A2)	0.1915	Agricultural production environment monitoring (B6)	0.3527
			Electrification in agricultural production (B7)	0.3936
			Total power of agricultural machinery (B8)	0.2536
	Rural commodity circulation digitalization (A3)	0.1730	Rural postal and communication service level (B9)	0.2370
			Rural consumer goods retail level (B10)	0.2518
			Length of rural postal delivery routes (B11)	0.2977
			Proportion of administrative villages with postal service (B12)	0.2135
	Rural marketing digitalization (A4)	0.1808	Number of comprehensive demonstration counties for e-commerce in rural areas (B13)	0.2888
			Number of Taobao villages (B14)	0.3290
			Rural e-commerce sales (B15)	0.3823
	Rural life service digitalization (A5)	0.1655	Level of inclusive financial services in rural areas (B16)	0.3465
			Expenditure on transportation and communication by rural households (B17)	0.2891
			Rural mobile payment index (B81)	0.3645
	Rural digitalization development potential (A6)	0.1297	Educational level of rural residents (B19)	0.2221
			Number of agricultural science and technology patents (B20)	0.2375
			Proportion of agricultural, forestry, and water financial expenditures (B21)	0.1583
			Per capita net income of rural households (B22)	0.1904
			Night time light intensity of rural areas (B23)	0.1918

The data in this study covers 330 samples from 30 provinces from 2011 to 2021 (excluding Tibet, Hong Kong, Macao, and Taiwan). The data of night-time light intensity data were derived from DMSP/OLS and NPP/VIIR datasets, adjusted and compiled to create continuous night-time light data spanning from 2000 to 2021. Due to the ’saturation’ and ’overflow’ effects of night-time light and its resolution limitations, using night-time light data to delineate rural and urban areas may encounter certain challenges [33]. Night-time light intensity is believed to be closely related to gross domestic product (GDP) [34]. Hence, Drawing on the work of Jing et al. [35], we obtained the annual nighttime light intensity of rural areas for each province by multiplying its ratio of annual added value of the primary industry to GDP by the total nighttime lights observed in each province for the respective year. Other data used were collected from China Statistical Yearbook, China Rural Statistical Yearbook, China Population and Employment Statistics Yearbook, Peking University digital inclusive finance index, provincial statistical yearbooks, provincial statistical bulletins, Ministry of Commerce website, etc. For missing data, mainly due to the lack of updates or changes in indicators, we obtain them by methods of linear interpolation.

## Results and analysis

### Evaluation results of China’s DVC

After inviting experts in the relevant field to score all primary and secondary indicators in the weight questionnaire through pairwise comparison, the obtained comparison data are then converted into intuitive fuzzy values according to the criteria for intuitive fuzzy value (Table 2). In this way, we obtained the intuitive fuzzy judgment matrices of the primary indicators and the secondary indicators: $R^{q}={\left(r_{gh}^{q}\right)}_{n\times n}={\left(t_{gh}^{q},f_{gh}^{q}\right)}_{n\times n}$, where *q* represents the ordinal number of the fuzzy judgment matrices and 1≤*q*≤7.

**Table 2:** Evaluation results of China’s DVC.

Region	Province	2011	2012	2013	2014	2015	2016	2017	2018	2019	2020	2021	Average
Eastern region	Beijing	0.420	0.430	0.431	0.444	0.453	0.482	0.481	0.481	0.497	0.500	0.554	0.470
	Tianjin	0.402	0.404	0.431	0.413	0.420	0.437	0.453	0.440	0.443	0.448	0.472	0.433
	Hebei	0.480	0.479	0.480	0.488	0.493	0.489	0.510	0.513	0.516	0.523	0.542	0.501
	Liaoning	0.408	0.400	0.409	0.420	0.440	0.454	0.457	0.462	0.467	0.472	0.490	0.443
	Shanghai	0.409	0.422	0.411	0.423	0.433	0.448	0.457	0.467	0.478	0.494	0.563	0.455
	Jiangsu	0.440	0.455	0.457	0.466	0.474	0.490	0.508	0.530	0.550	0.557	0.622	0.504
	Zhejiang	0.434	0.443	0.452	0.457	0.475	0.493	0.504	0.507	0.527	0.540	0.618	0.495
	Fujian	0.415	0.415	0.418	0.420	0.436	0.435	0.447	0.452	0.467	0.467	0.505	0.443
	Shandong	0.477	0.484	0.492	0.504	0.510	0.510	0.530	0.541	0.548	0.558	0.597	0.523
	Guangdong	0.436	0.436	0.444	0.447	0.461	0.474	0.497	0.523	0.554	0.562	0.631	0.497
	Guangxi	0.419	0.418	0.423	0.423	0.433	0.449	0.464	0.469	0.472	0.476	0.501	0.450
	Hainan	0.444	0.437	0.440	0.438	0.442	0.443	0.453	0.459	0.465	0.466	0.502	0.454
Central region	Shanxi	0.424	0.425	0.427	0.423	0.433	0.443	0.451	0.460	0.453	0.459	0.481	0.444
	Inner Mongolia	0.408	0.412	0.415	0.423	0.441	0.436	0.462	0.469	0.474	0.470	0.495	0.446
	Jilin	0.413	0.419	0.422	0.420	0.435	0.442	0.445	0.455	0.456	0.468	0.479	0.441
	Heilongjiang	0.421	0.429	0.440	0.438	0.450	0.465	0.476	0.470	0.485	0.487	0.512	0.461
	Anhui	0.413	0.428	0.425	0.436	0.446	0.455	0.468	0.478	0.476	0.481	0.514	0.456
	Jiangxi	0.414	0.418	0.416	0.421	0.431	0.433	0.446	0.450	0.455	0.464	0.498	0.440
	Henan	0.448	0.456	0.456	0.465	0.471	0.475	0.490	0.490	0.511	0.514	0.562	0.485
	Hubei	0.427	0.431	0.432	0.437	0.449	0.456	0.465	0.479	0.492	0.503	0.527	0.464
	Hunan	0.416	0.424	0.424	0.419	0.432	0.445	0.467	0.471	0.477	0.488	0.511	0.452
Western region	Sichuan	0.421	0.423	0.424	0.433	0.445	0.463	0.482	0.507	0.496	0.494	0.525	0.465
	Guizhou	0.383	0.388	0.391	0.411	0.424	0.449	0.460	0.465	0.470	0.467	0.487	0.436
	Yunnan	0.410	0.413	0.419	0.424	0.433	0.446	0.488	0.479	0.477	0.475	0.500	0.451
	Shannxi	0.412	0.414	0.418	0.418	0.432	0.440	0.450	0.456	0.452	0.463	0.489	0.440
	Gansu	0.414	0.422	0.424	0.427	0.439	0.462	0.454	0.469	0.464	0.469	0.490	0.448
	Qinghai	0.394	0.388	0.392	0.416	0.420	0.451	0.451	0.467	0.468	0.472	0.483	0.436
	Ningxia	0.377	0.399	0.407	0.406	0.415	0.426	0.443	0.443	0.447	0.463	0.478	0.428
	Xinjiang	0.405	0.410	0.408	0.419	0.429	0.452	0.454	0.448	0.461	0.469	0.484	0.440
	Chongqing	0.364	0.386	0.392	0.398	0.408	0.412	0.427	0.434	0.449	0.448	0.470	0.417

Subsequently, consistency verification of the judgment matrixes was conducted based on Formula (1). When σ is set to 0.5, all the *τ* values of judgment matrices that originally failed the consistency test were less than 0.1, which suggests all adjusted judgment matrices (${\tilde{R}}^{q}={\left({\tilde{r}}_{gh}^{q}\right)}_{n\times n}={\left({\tilde{t}}_{gh}^{q},{\tilde{f}}_{gh}^{q}\right)}_{n\times n}$) have passed the consistency test.

After calculating the matrices using Formula (3) and (4), we obtained the normalized weights for all primary and secondary indicators (Table 1). The combination weights of B1-B22 are 0.0443, 0.0386, 0.0314, 0.0257, 0.0196, 0.0676, 0.0754, 0.0486, 0.0410, 0.0435, 0.0515, 0.0369, 0.0522, 0.0595, 0.0691, 0.0573, 0.0478, 0.0603, 0.0288, 0.0308, 0.0205, 0.0247, 0.0249 respectively.

After the evaluation index system had been constructed and all weights of primary indicators and secondary indicators were acquired, we collected data on DVC of the 30 provinces in China. Upon calculation using Formula (7,8), we obtained the evaluation results of the target regions’ DVC during the period from 2011 to 2021 (Table 2).

### Temporal analysis of China’s DVC

From Table 2, it can be observed that the DVC index in China has exhibited a gradual increase from 2011 to 2021. The national average of the DVC index increased from 0.418 in 2011 to 0.519 in 2021. The average annual growth rate for each year is 2.20%. The year 2021 witnessed the most rapid growth in the DVC index value, experiencing a remarkable growth rate of 6.59%. On the other hand, 2013 marked the year with the slowest growth, as the index only increased by 0.004, corresponding to a modest growth rate of 0.90%. The average DVC index for all regions and all years is 0.457. The mean values for all provinces in all recorded years are between 0.4 and 0.6, which indicates that the overall level of DVC in China is still relatively low, and there is still significant room for improvement. The top five provinces by DVC index averages are Shandong, Jiangsu, Hebei, Guangdong, and Zhejiang while the bottom five are Chongqing, Ningxia, Tianjin, Guizhou, and Qinghai.

From a temporal perspective, the DVC indexes of all provinces have exhibited a marked growth trend, despite occasional declines in some years. In 2014, 2019, and 2020, there was a relatively high number of provinces where the DVC index declined. In 2014, eight provinces experienced a decrease in their DVC indexes, namely Tianjin, Hainan, Shanxi, Jilin, Heilongjiang, Hunan, Shaanxi, and Ningxia. Similarly, in 2019, six provinces, including Shanxi, Anhui, Sichuan, Yunnan, Shaanxi, and Gansu, observed a decline in the DVC index. In 2020, five provinces, namely Fujian, Inner Mongolia, Sichuan, Guizhou, Yunnan, and Chongqing, also witnessed a decrease in their DVC indexes.

We also found that during the sample period, the eastern region had the largest internal regional variance, with a variance value of the DVC index for all years at 0.047. In comparison, the variance in the central and western regions is 0.030 and 0.032 respectively across all years. Additionally, the variance in DVC indexes of the eastern provinces has shown an increasing trend over the sample period.

We classified China’s DVC index for 2011 and 2021 using the natural breaks method, and the results are shown in Table 3. In 2011, the regions with high to upper-middle levels of DVC were mainly concentrated in the eastern and central parts of the country. By 2021, the three provinces of Jiangsu, Zhejiang, and Guangdong, which were previously at the upper-middle level in 2011, were transitioned to the high-level group. Comparatively, Hebei, which was in the high-level category in 2011, dropped to the upper-middle level by 2021. In 2011, there were three provinces at the low level: Chongqing, Ningxia, Guizhou. By 2021, the number of provinces at the low level had increased to nine, including Chongqing, Tianjin, Ningxia, Jilin, among others. It is worth noting that the range for high-level DVC in 2011 was between 0.451 and 0.480, lower than the range for the low-level category in 2021, which spanned from 0.470 to 0.490. This shows that the overall DVC levels in China in 2021 were much higher than those in 2011.

**Table 3:** Members of cluster types of China’s DVC index in 2011 and 2021.

2011			2021		
Cluster types	Range of index	Members	Cluster types	Range of index	Members
Low level	0.360~0.390	Chongqing, Ningxia, Guizhou	Low level	0.470~0.490	Chongqing, Tianjin, Ningxia, Jilin, Shanxi, Xinjiang, Qinghai, Guizhou, Shannxi
Lower-middle level	0.391~0.420	Qinghai, Tianjin, Xinjiang, Liaoning, Inner Mongolia, Yunnan, Anhui, Shanghai, Shannxi, Jilin, Gansu, Fujian, Jiangxi, Hunan, Guangxi	Lower-middle level	0.491~0.530	Liaoning, Gansu, Inner Mongolia, Jiangxi, Yunnan, Guangxi, Hainan, Fujian, Heilongjiang, Anhui, Hunan, Sichuan, Hubei
Upper-middel level	0.421~0.450	Heilongjiang, Sichuan, Beijing, Shanxi, Hubei, Zhejiang, Guangdong, Jiangsu, Henan, Hainan	Upper-middel level	0.531~0.590	Hebei, Henan, Beijing, Shanghai
High level	0.451~0.480	Shandong, Hebei	High level	0.591~0.630	Shandong, Zhejiang, Jiangsu, Guangdong

**Fig 1:**
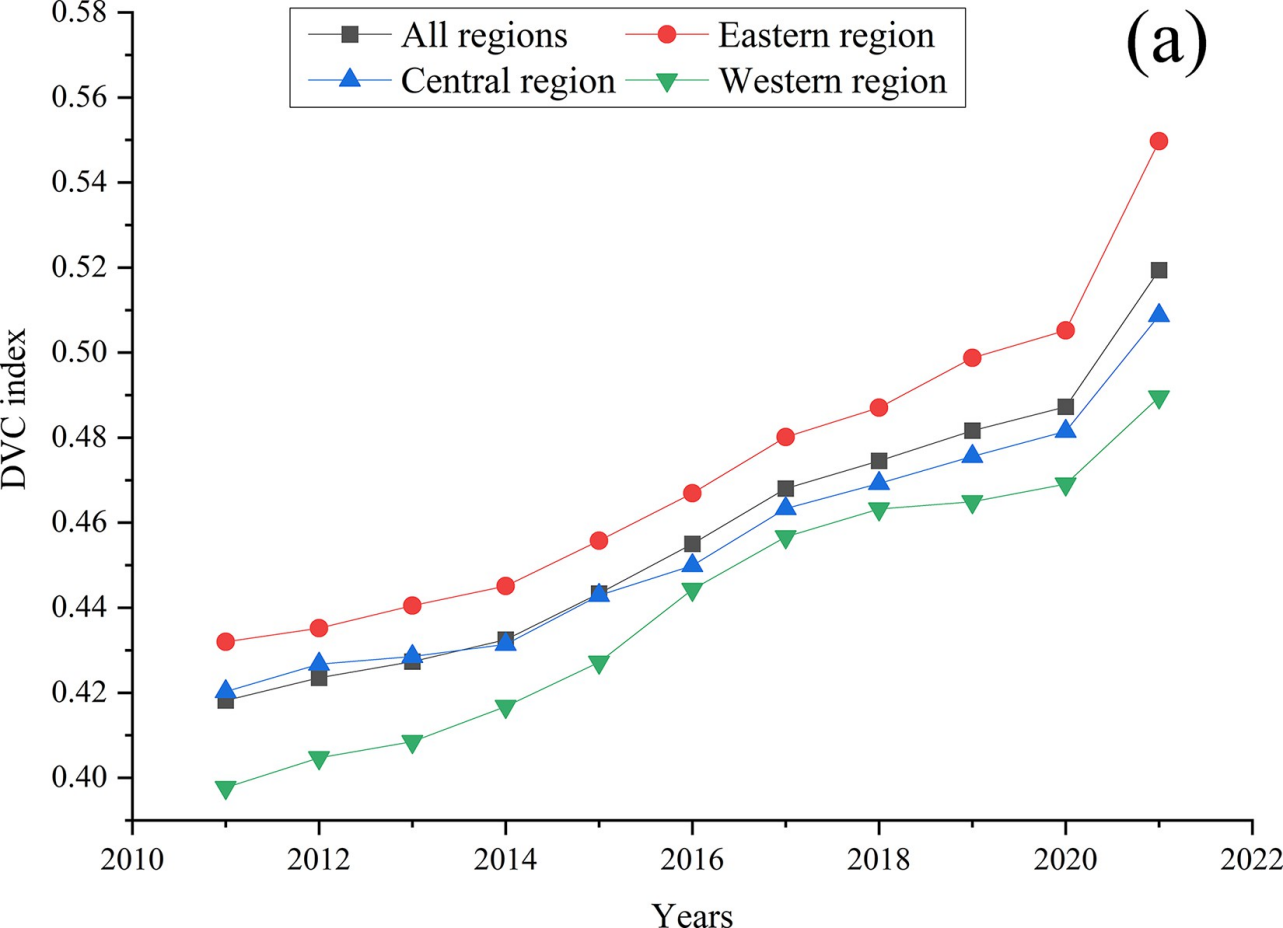
Temporal evolution of the DVC index of China from 2011 to 2021.

Fig 1 provides a visual depiction of the overall DVC index of eastern, central, and western regions of China from 2011 to 2021. From Fig 1, it can be seen that, in general, the DVC indexes of China’s three regions show upward trends every year, with the fastest growth in 2021. Specifically, the DVC index of the eastern region has been higher than those of the central and western regions in all years, while the index in the western region has been lower than that in the eastern and central regions in all years. This reflects the uneven nature of DVC among the eastern, central, and western regions of China. The average DVC index of all regions nationwide is equal to or slightly higher than the level of the central region. The variances between the eastern and western areas tended to exhibit a trend of initially narrowing and then expanding. Specifically, we observed that the gap between the western and eastern regions was smallest in 2017 and largest in 2021.

**Fig 2:**
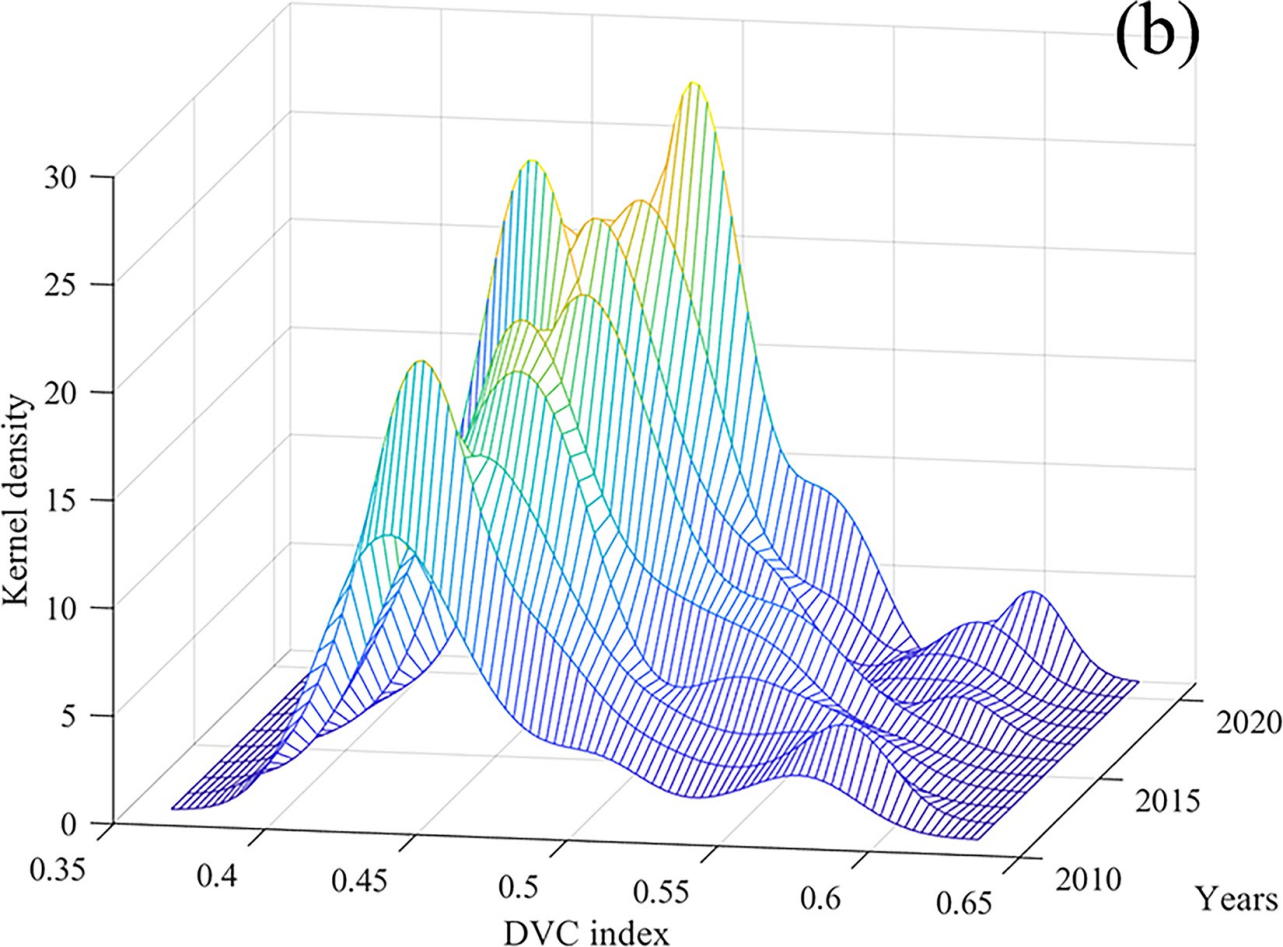
Kernel density curve of the DVC index of China from 2011 to 2021.

To observe the variances and dynamic evolution patterns of DVC in these regions more clearly, we utilized Matlab 2022a and applied the kernel density estimation method to analyze their status, flexibility, and distribution. The results are depicted in Fig 2. In the figure, the positions of the curve distribution indicate the levels of DVC, where high positions signify high digital rural construction. The width and height of the peaks signify the scale of regional disparities, while the quantity of peaks indicates the degree of polarization [36]. The spread of the curve distribution mirrors the spatial variations between the area exhibiting the highest degree of DVC and other areas. The extent of these variations correlates with the length of the curve tails [36]. Fig 2 depicts the kernel density plot for China’s DVC from 2011 to 2021. Overall, the main peak is gradually shifting to the right, indicating that the level of China’s DVC is showing an upward trend year by year. The peaks’ positions were skewed to the left, signifying that the overall levels of DVC in China are not very high. Compared to other years, the main peaks in 2018 was particularly skewed to the left, indicating a slowdown in the overall growth trend of China’s DVC during the year. In 2011, 2017, 2018, and 2021, the kurtosis was relatively high, indicating that the absolute gap in the DVC indexes of all provinces narrowed, and the degree of dispersion was decreasing. During the sample period, the kernel density estimation curve exhibited a clear unimodal pattern, indicating that for most years, there was no significant multipolar differentiation. However, there were noticeable fluctuations on the right side of the curve in 2012, 2017, 2020, and 2021, indicating a mild polarization trend in these years. All annual density curves exhibit a pronounced right-skewed tail, with no reduction in tail width, suggesting a certain level of disparity in the DVC index between regions in China, and this disparity has not shown a decreasing trend over time.

### Spatial network structure of China’s DVC

We employed Formula (9) to construct the gravity matrix for the DVC index of the 30 provinces in China. Subsequently, we created the spatial binary matrix to analyze the spatial correlation of DVC in China using the aforementioned method. Then we conducted a comprehensive analysis of the whole network characteristics, individual network characteristics, and spatial clustering characteristics using Ucinet 6 software.

#### Whole network structure characteristics of China’s DVC

After analyzing the overall density of the spatial binary matrix, we found that the number of ties in China’s DVC spatial correlation network was 180 in 2011, which then increased to 181 in 2016 and further rose to 182 in 2021. Overall, the number of ties of China’s DVC spatial correlation network showed a slow growth trend during the sample period. However, the overall number of ties is still suboptimal, with a considerable gap from the maximum value of 870 (29×30) [37]. This indicates that there is significant room for improvement in the overall number of ties in the spatial correlation network. In other words, the spatial association among provinces necessitates reinforcement. We also observed that the network density of China’s DVC has been increasing. In 2011, the network density was 0.207, which rose to 0.208 in 2016 and further increased to 0.209 in 2018. Additionally, throughout the sample period, network connectedness within China’s DVC spatial correlation network consistently remained at 1. This stability arises from the absence of unreachable pairs, providing compelling evidence of significant spatial spillover effects of the network [38].

Analysis using the Krackhardt GTD method revealed that the hierarchical level of the network has remained stable at around 0.345 since 2011, indicating strong mutual influences among provinces in China’s DVC spatial correlation network, and a relatively stable network structure. The network efficiency for each year has also been relatively stable, with values fluctuating around 0.71. The lowest value was in 2018 at 0.7118, and the highest was in 2021 at 0.7176. This indicates that the spatial correlation network of China’s DVC demonstrates comparatively high network efficiency.

#### Individual network structure characteristics of China’s DVC

The individual network structure characteristics of the spatial correlation network for DVC in 30 provinces of China are shown in Table 4. Degree centrality quantifies the extent to which a node is linked to other nodes within the network [26]. As can be seen in Table 4, the top five provinces in China in terms of outdegree centrality are Shanghai, Beijing, Tianjin, Jiangsu, and Zhejiang. These provinces are all located in the eastern region of China and are also the most economically developed areas in China. Additionally, they are technologically advanced, with higher levels of education among residents, convenient transportation, and well-established digital infrastructure. Therefore, these provinces are more likely to have spatial correlations with other provinces, playing crucial roles in the development of the entire DVC spatial correlation network. In terms of indegree centrality, the top five provinces in China are Guizhou, Sichuan, Gansu, Chongqing, and Hubei. All these provinces are in the central and western regions of China. To support the construction of digital villages in these regions, the central government has allocated significant funds, manpower, and other resources. In other words, these areas benefit from an influx of economic, technological, and cultural elements, and occupy relatively important positions in the DVC spatial correlation network.

**Table 4:** Centrality analysis of spatial correlation networks of China’s DVC.

Sort	Degree centrality					Closeness centrality				Middle centrality	
	Outdegree		Indegree			Incloseness		Outcloseness			
1	Shanghai	26	Guizhou	9	Heilongjiang		17.16	Shanghai	87.879	Beijing	88.173
2	Beijing	24	Sichuan	9	Yunnan		15.026	Tianjin	85.294	Guangdong	74.767
3	Tianjin	24	Gansu	8	Jilin		14.573	Beijing	85.294	Tianjin	69.757
4	Jiangsu	22	Chongqing	8	Liaoning		14.573	Jiangsu	78.378	Gansu	66
5	Zhejiang	17	Hubei	8	Qinghai		14.216	Zhejiang	67.442	Shanghai	59.831
6	Fujian	9	Yunnan	8	Sichuan		13.303	Henan	54.717	Guizhou	58.702
7	Guangdong	7	Guangdong	7	Guizhou		13.242	Anhui	54.717	Chongqing	48.667
8	Anhui	6	Jiangxi	7	Chongqing		13.122	Jiangxi	50.877	Jiangxi	46.919
9	Gansu	6	Shandong	7	Hubei		13.063	Shandong	50	Fujian	46.767
10	Henan	5	Guangxi	7	Gansu		12.775	Hebei	49.153	Jiangsu	35.929
11	Jiangxi	5	Hunan	7	Xinjiang		12.775	Shanxi	47.541	Zhejiang	29.993
12	Shandong	3	Hainan	7	Guangdong		12.664	Inner Mongolia	47.541	Henan	12.984
13	Guizhou	4	Fujian	6	Guangxi		12.554	Guangdong	40.845	Hubei	12.25
14	Hebei	4	Henan	6	Hunan		12.554	Fujian	40.845	Anhui	10.769
15	Chongqing	4	Shannxi	6	Jiangxi		12.554	Guizhou	32.955	Shandong	9.602
16	Shanxi	2	Heilongjiang	6	Fujian		12.554	Guangxi	29.897	Shanxi	4.233
17	Guangxi	2	Qinghai	6	Hainan		12.554	Hunan	29.897	Hebei	4
18	Hunan	2	Shanghai	5	Shaanxi		12.5	Hainan	29.293	Guangxi	3.802
19	Liaoning	2	Beijing	5	Ningxia		12.446	Chongqing	27.885	Hunan	3.802
20	Inner Mongolia	2	Tianjin	5	Shanghai		11.789	Hubei	25	Liaoning	3
21	Hainan	1	Anhui	5	Zhejiang		11.741	Gansu	23.577	Inner Mongolia	2.833
22	Hubei	1	Liaoning	5	Jiangsu		11.647	Sichuan	21.97	Sichuan	2.4
23	Jilin	1	Jilin	5	Shandong		11.462	Shaanxi	19.205	Hainan	1.819
24	Ningxia	1	Ningxia	5	Henan		11.417	Ningxia	19.205	Shaanxi	1
25	Shannxi	1	Xinjiang	5	Anhui		11.373	Liaoning	3.571	Ningxia	1
26	Sichuan	1	Zhejiang	4	Shanxi		11.328	Jilin	3.567	Jilin	1
27	Yunnan	0	Shanxi	4	Inner Mongolia		11.328	Heilongjiang	3.333	Yunnan	0
28	Heilongjiang	0	Inner Mongolia	4	Tianjin		10.821	Yunnan	3.333	Qinghai	0
29	Qinghai	0	Jiangsu	3	Beijing		10.781	Qinghai	3.333	Xinjiang	0
30	Xinjiang	0	Hebei	2	Hebei		10.069	Xinjiang	3.333	Heilongjiang	0

Closeness centrality gauges a node’s proximity to all other nodes within the network by assessing the average shortest path length from that node to all others 26. The five provinces with the highest incloseness centrality are Heilongjiang, Yunnan, Jilin, Liaoning, and Qinghai. All these provinces have relatively convenient transportation and have good channels for exchanging information and technology with surrounding provinces. Therefore, they are likely to establish connections with other provinces during the process of DVC and are relatively closer to the central positions in the network. The top five provinces with the highest outcloseness to centrality are Shanghai, Tianjin, Beijing, Jiangsu, Zhejiang. These areas are relatively more developed in terms of economy, culture, education, and technology. Their advanced transportation and information distribution systems, enable them to establish connections with other cities more effortlessly during the digital village development process. This facilitates the generation of spillover effects with surrounding provinces, positioning them closer to central positions within the spatial correlation network.

Middle centrality indicates the degree of a node acting as a bridge, connecting two other nodes via the shortest path. According to Table 4, the top five provinces in China in terms of outdegree centrality are Beijing, Guangdong, Tianjin, Gansu, and Shanghai. A shared trait among these regions is their role as regional hubs, possessing robust capabilities to coordinate and synergize development with other provinces, and serving as crucial intermediaries and facilitators. Beijing, Guangdong, Tianjin, and Shanghai are situated in the eastern part of China and are at the forefront of digital village development nationwide, thus naturally playing significant bridging roles in the spatial correlation network of DVC in China. Despite being a western province with an underdeveloped economy, culture, and technology, Gansu boasts a relatively sound transportation infrastructure, enabling it to wield influence over neighboring provinces.

### Spatial clustering characteristics of China’s DVC

We utilized the block-modeling approach, specifically the Convergent Correlations (CONCOR) technique, to examine the spatial clustering within the spatial correlation network of China’s DVC. When the max depth of splits was set to 2, and the convergence criteria was set to 0.2, the correlation network of China’s DVC was divided into four blocks (Table 5). Members of Block I and Block II are predominantly situated in the central and western regions of China, whereas members of Block III and Block IV are all located in the eastern part of China.

**Table 5:** Division of spatial correlation blocks in China’s DVC.

Block	Province
Block Ⅰ	Anhui、Jiangxi、Sichuan、Gansu、Qinghai、Guangxi、Guizhou、Hainan、Shannxi、Hunan、Heilongjiang、Hubei、Ningxia、Yunnan、Chongqing、Xinjiang
Block Ⅱ	Inner Mongolia、Henan、Liaoning、Hebei、Jilin、Shanxi、Shandong
Block Ⅲ	Fujian、Guangdong
Block Ⅳ	Beijing、Shanghai、Tianjin、Jiangsu、Zhejiang

As depicted in Table 6, in the four blocks in the spatial correlation network of DVC in China, there exist evident spatial clustering effects and spatial spillover phenomena. The total number of relationships is 181. Of these, 32 are internal relationships, representing 17.7% of the total network relationships. External relationships between the blocks total 149, making up 82.3% of the total network relationships. Block Ⅰ, consisting of 16 provinces, has 17 internal contacts received, 89 external contacts received, 18 contacts sent externally, and 17 contacts sent internally. The expected internal relationship ratio of Block Ⅰ is 51.72% and the actual internal relationship ratio of Block Ⅰ is 48.57%. The expected internal relationship ratio surpasses the actual internal relationship ratio, indicating that it is a main inflow block. Members of this block, compared to members of other blocks, receive more input from others during the process of digital village development. Block Ⅱ has 9 internal contacts received, 30 external contacts received, 12 contacts sent externally, and 9 contacts sent internally. The expected internal relationship ratio of Block Ⅱ is 24.14% and the actual internal relationship ratio of Block Ⅱ is 42.86%. This block not only sends relationships to other blocks but also receives relationships from other blocks, making it a bidirectional spillover block. Block Ⅲ consists of two provinces: Fujian and Guangdong. Block Ⅲ has 9 internal relationships within the block, receiving 30 contacts from outside, and sending out 12 contacts to outside blocks. Its expected internal relationship ratio is 3.45%, while the actual internal relationship ratio is 0%. Block III has relatively more contacts sent and received from outside than contacts sent and received from inside. Therefore, Block III is classified as an agent block. Block Ⅳ includes Beijing, Shanghai, Tianjin, Jiangsu, and Zhejiang, which are provinces relatively developed in terms of economy, culture, and technology. They possess robust software and hardware infrastructure conducive to the development of digital rural areas. The number of internal relations in Block IV is 6, while it receives 17 external relations and sends out 105 external relations. The expected internal relationship ratio is 13.79%, whereas the actual internal relationship ratio is 5.41%. Consequently, it is classified as a main outflow block.

**Table 6:** Spillover effects of spatial correlation blocks in China’s DVC.

Block	Contacts received			Contacts sent		Number of members	Expected internal relationship(%)	Actual internal relationship(%)	Block role
	Inside	Outside	Inside		Outside				
Block Ⅰ	17	89	17		18	16	51.72	48.57	main inflow
Block Ⅱ	9	30	9		12	8	24.14	42.86	bidirectional spillover
Block Ⅲ	0	13	0		14	2	3.45	0.00	agent
Block Ⅳ	6	17	6		105	5	13.79	5.41	main outflow

Drawing from the correlation distribution among the blocks as depicted in Table 6, we developed the density matrix and image matrix of spatial correlation blocks in China’s DVC (Table 7). If the density of a block exceeds the overall network density level of the spatial correlation network, which is 0.2, then this block exhibits a concentration trend, and it is assigned a value of 1; otherwise, it is assigned a value of 0. Following this rule, a matrix can be obtained. The image matrix provides a visual representation of the spillover relationships between different blocks in China’s digital rural development, offering a clear insight into the mutual influence mechanisms among provinces. In Table 7, it is evident that Block I exhibits a spillover relationship with Block III, Block II demonstrates a spillover relationship with Block IV, and Block III displays a spillover relationship with Block I. The self-spillover effects of Block I, Block II, and Block III are not significant, while Block IV exhibits a strong self-spillover effect. Specifically, there is a strong exchange and cooperation effect among the five members of Block IV, namely Beijing, Shanghai, Tianjin, Jiangsu, and Zhejiang. Additionally, they have exerted a certain influence on the DVC of members of the other three blocks. In contrast to members of Block IV, members of Block I, Block II, and Block III lack effective communication and interaction mechanisms internally.

**Table 7:** Density matrix and image matrix of spatial correlation blocks in China’s DVC.

Block	Density matrix				Image matrix			
	BlockⅠ	Block Ⅱ	Block Ⅲ	Block Ⅳ	BlockⅠ	Block Ⅱ	Block Ⅲ	Block Ⅳ
Ⅰ	0.067	0.009	0.344	0.075	0	0	1	0
Ⅱ	0.027	0.143	0	0.286	0	0	0	1
Ⅲ	0.438	0	0	0	1	0	0	0
Ⅳ	0.988	0.743	0.2	0.3	1	1	1	1

### Driving mechanism

Drawing from existing literature [39–41], this study identifies six driving factors influencing the construction of digital village in China, including level of social consumption, level of scientific and educational development, level of economic development, investment in information infrastructure, fiscal expenditure on education, and transportation infrastructure. Level of social consumption is derived by dividing the total retail sales of social consumer goods by the corresponding region’s GDP of each province. Level of scientific and educational development is represented by the average years of education received per person within a given area. Level of economic development is represented by the per capita GDP in the given province/municipalities. Data for investment in information infrastructure is represented by fixed asset investment in information transmission, computer services, and software industry per capita in each area. Fiscal expenditure on education is calculated by dividing the per capita educational expenditure by the per capita gross domestic product. Transportation infrastructure is measured by the mileage of roads within a given area. Geodetector was utilized to probe these factors, analyzing their impacts on the spatial differentiation of DVC in China, pinpointing the primary driving factors, and exploring the mechanisms of interaction among these factors.

**Fig 3:**
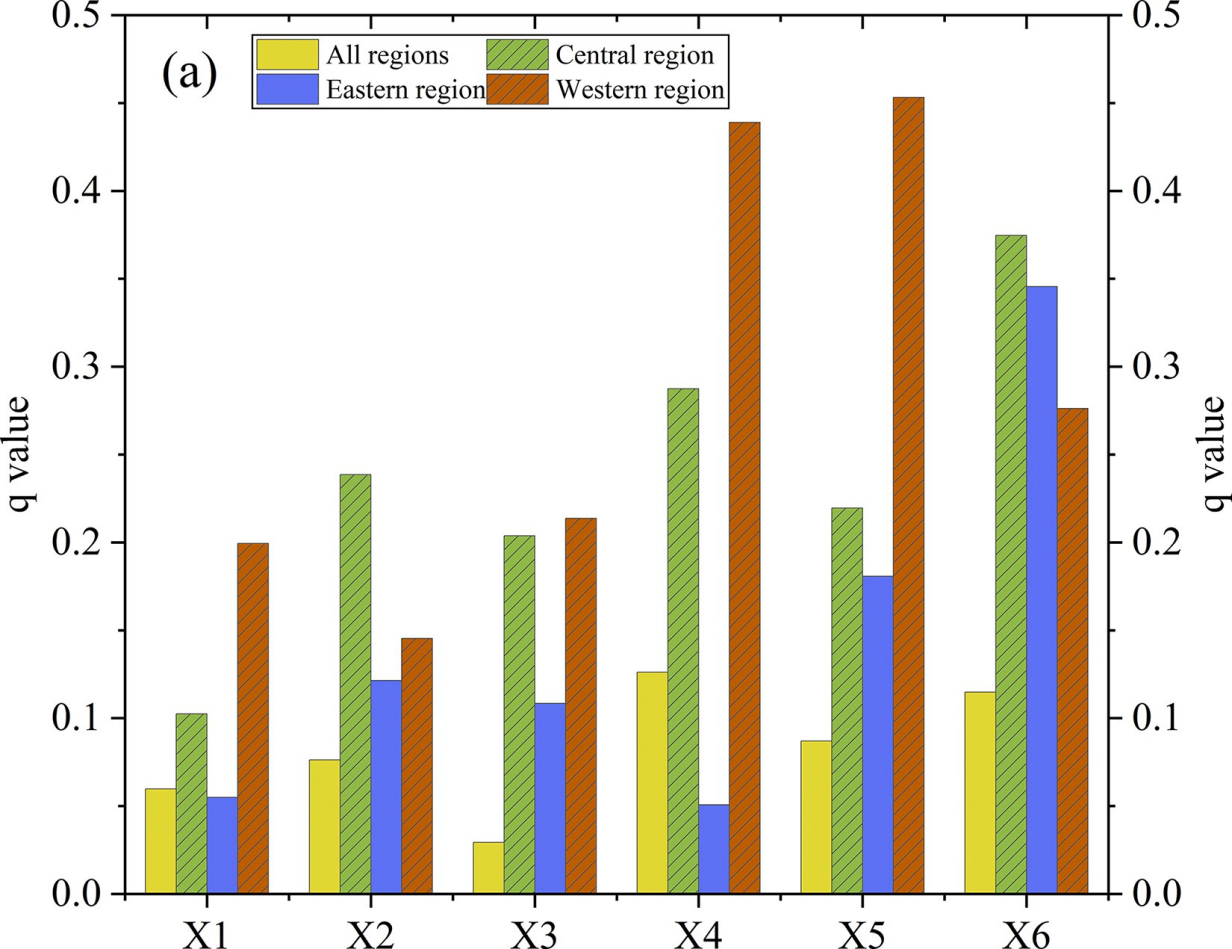
Q-values of driving factors. Note: X1 = level of social consumption, X2 = level of scientific and educational development, X3 = level of economic development, X4 = investment in information infrastructure, X5 = fiscal expenditure on education, X6 = transportation infrastructure.

The q-value obtained through Geodetector quantifies the degree to which each driving factor affects the spatial differentiation of DVC in China. A higher q-value of a driving factor indicates a stronger impact of the factor on the spatial differentiation of DVC in China. From Fig 3, it can be observed that across all regions, the factors with the greatest influence on the DVC index are investment in information infrastructure and transportation infrastructure, both with q-values exceeding 0.1. The q-values for level of scientific and educational development and fiscal expenditure on education are both greater than 0.075. The q-values for the level of social consumption and level of economic development are 0.06 and 0.029, respectively. It’s worth noting that, apart from the p-value of level of economic development being less than 0.05, the p-values of the other five driving factors are all less than 0.001. For the eastern region, the main driving factors of DVC in China are level of scientific and educational development, level of economic development, fiscal education expenditure, and transportation infrastructure, all of which have q-values exceeding 0.1. The q-values for level of social consumption and investment in information infrastructure are 0.055 and 0.051, respectively. Apart from that of investment in information infrastructure, the p-values for all six driving factors are significant (< 0.05). For the central region, all six driving factors have significant impacts on the level of digital village development, with values exceeding 0.1. Specifically, the q-values for level of scientific and educational development, investment in information infrastructure, and transportation infrastructure are all greater than 0.23. It’s worth noting that the p-value for level of social consumption is 0.145, exceeding the threshold of 0.1, indicating insignificance. For the western region, all six driving factors have significant impacts on the level of digital rural development, with values exceeding 0.15, and all p-values are less than 0.05. Specifically, the q-values for investment in information infrastructure and fiscal expenditure on education are both greater than 0.4, indicating that these variables have relatively strong explanatory power.

**Fig 4:**
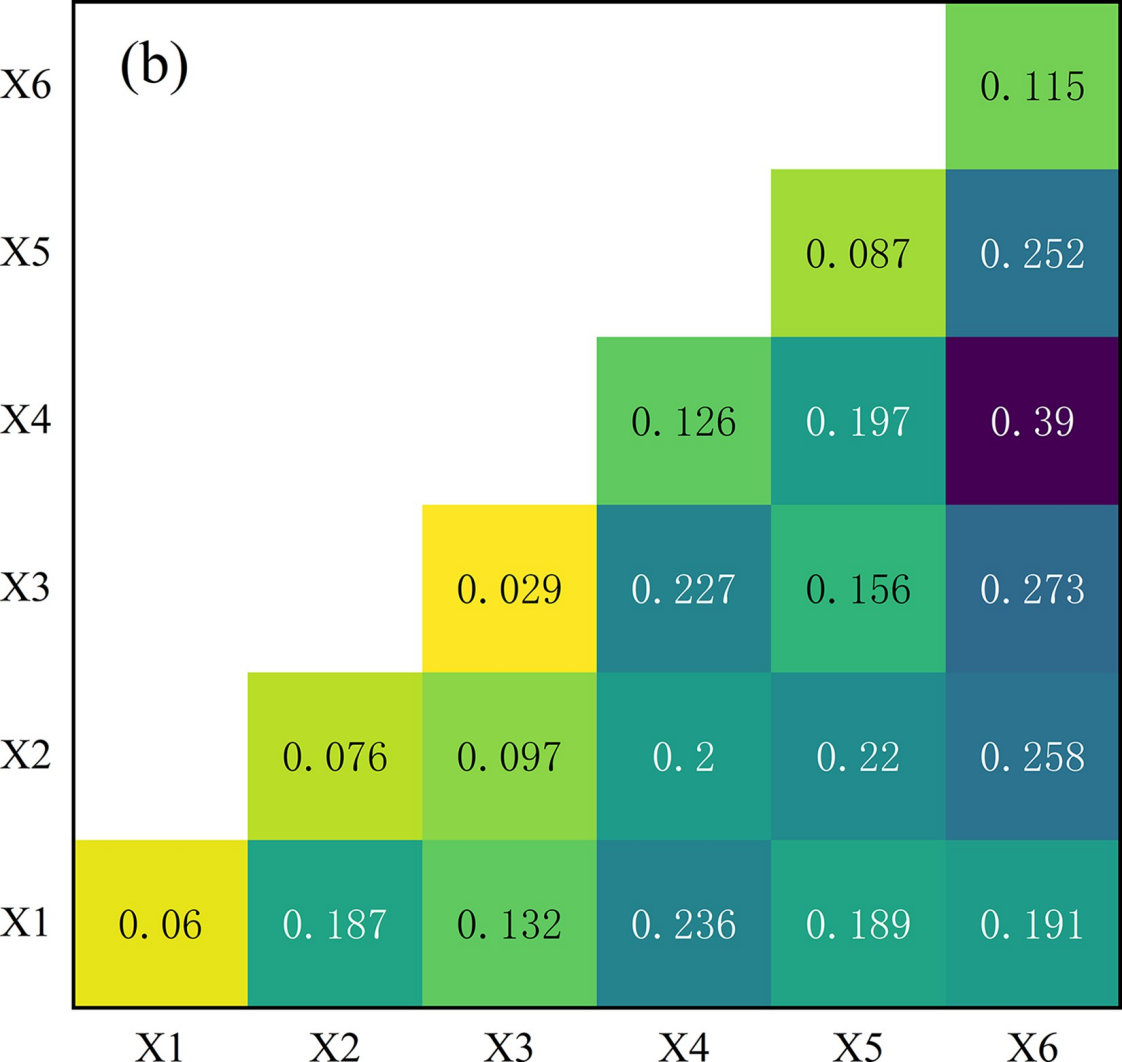
The interaction detector results. Note: X1 = level of social consumption, X2 = level of scientific and educational development, X3 = level of economic development, X4 = investment in information infrastructure, X5 = fiscal expenditure on education, X6 = transportation infrastructure.

To further explore the explanatory power of interaction among different driving factors on the differentiation of DVC in China, we used the method of interaction detector to analyze the data of DVC from all regions. The results are depicted in Fig 4. As evident from the charts, all the q-values of interaction surpass those of individual factors, suggesting that there is a strengthening effect in the interactions between all pairs of influencing factors. Each pair of factors exhibits either bivariate or non-linear enhancement in explaining the spatial variation of DVC in China. Specifically, the interaction between X2 and X3 (X2∩X3) demonstrates a bivariate enhancement effect, as does the interaction between X2 and X4 (X2∩X4). In essence, the explanatory power of the interaction between these two factors exceeds that of individual factors but falls short of the combined explanatory power of these two individual factors 36–40 [42]. All other interaction effects belong to nonlinear enhancement, indicating that the explanatory power of the interaction between two factors exceeds the combined explanatory power of these two individual factors [42]. This suggests that the cumulative effect of the interaction of these driving factors strengthens the spatial variation of DVC in China. As shown in the charts, the interaction effects of X4, X5, and X6 with other factors exhibit strong explanatory power. Specifically, the interaction between X4 and X1, X2, X3, the interaction between X5 and X2, X4, and the interaction between X6 and X2, X3, X4, and X5 all have a significant influence on the spatial variation of DVC in China.

## Conclusions and policy recommendations

### Conclusions

DVC has the potential to drive rural development by bridging the urban-rural information gap, modernizing traditional agricultural practices, enhancing employment prospects for rural dwellers, mitigating income inequality, and enhancing rural ecological conditions. In China, there is notable regional diversity regarding resources for DVC, such as digital infrastructure and financial investment, and the level of DVC varies across different regions. Evaluating the current state of DVC across different Chinese regions, elucidating the temporal and spatial dynamics of China’s DVC, and pinpointing the specific factors propelling China’s DVC are essential for developing strategies to enhance future DVC in China and for addressing the regional imbalances in the development levels of digital villages.

This study first established a comprehensive evaluation index system for DVC in China, calculated the weights of indicators using IFAHP, and computed the DVC index for the 30 provinces in China from 2011 to 2021 using the dynamic GRA method. Subsequently, the dynamic structure of DVC was analyzed through temporal evolution analysis and Kernel Density Curve analysis. Furthermore, based on the DVC index values, the spatial network structure of China’s DVC was analyzed using UCINET 6 software. Lastly, the driving factors causing spatial differentiation in China’s DVC construction were explored using Geodetector.

The development of digital villages in China from 2011 to 2021 has consistently demonstrated progressive enhancement. The eastern region leads in DVC development, maintaining an average index of 0.472 across all years. The central region holds a second position with an average DVC index of 0.454, and the western region ranks lowest with an average index of 0.440. The average DVC index stands at 0.457 for all regions throughout the years surveyed. This suggests that the DVC in China, while on an upward trajectory, remains at a nascent stage with ample potential for improvement. Furthermore, we found that compared to the central and western regions, the eastern region has the largest internal regional variance during the sample period, and this variance shows an increasing trend over time.

The dynamic trend analysis reveals that Jiangsu, Zhejiang, and Guangdong have made significant progress in digital rural development. The three provinces, which were previously at the upper-middle-level category in 2011, were transitioned to the high-level category by 2021. In 2011, there were three provinces at the low level. By 2021, the number of provinces at the low level increased to nine. It’s noteworthy that in 2011, the high-level category range [0.451, 0.480] was lower than the low-level category range [0.470, 0.490] in 2021. The kernel density estimation analysis demonstrates that in 2018, the predominant peaks were noticeably skewed towards the left, suggesting a slowdown in the overall growth trajectory. The kernel density curve across all sample years displayed a distinct unimodal pattern, suggesting that there was no notable multipolar differentiation. All yearly density curves display a prominent right-skewed tail, with no decrease in tail width, indicating a consistent level of disparity in the DVC index among regions in China and the inequality has not exhibited a declining trend over time.

The overall network structure analysis suggests that the number of ties in China’s DVC spatial correlation network grew slowly but still falls significantly short of the ideal number. We also noted an increase in the network density of China’s DVC, providing strong evidence of spatial spillover effects within the network. The analysis of individual network structures reveals that Shanghai, Beijing, Tianjin, Jiangsu, and Zhejiang are the top five provinces in China in terms of outdegree centrality. This implies their stronger spatial correlations with other provinces. Regarding indegree centrality, Guizhou, Sichuan, Gansu, Chongqing, and Hubei emerge as the top five provinces in China, which indicates they benefit much from economic, technological, and cultural inputs from others and hold relatively significant positions within the DVC spatial correlation network. The five provinces with the highest incloseness centrality are Heilongjiang, Yunnan, Jilin, Liaoning, and Qinghai, which suggests they can establish connections with other cities effortlessly and have relatively easier access to elements necessary for DVC. The top five provinces with the highest outcloseness centrality are Shanghai, Tianjin, Beijing, Jiangsu, and Zhejiang. They have a greater capacity to generate spatial spillovers to other provinces, positioning them closer to central positions within the network. In terms of outdegree centrality, the top five provinces in China are Beijing, Guangdong, Tianjin, Gansu, and Shanghai, which means they play significant bridging roles in the spatial correlation network.

Geodetector analysis indicates that the driving factors exerting the most significant influence on the level of DVC in China include investment in information infrastructure, fiscal expenditure on education and transportation infrastructure, all q-values surpassing 0.08. In the eastern region, pivotal drivers comprise levels of scientific and educational development, fiscal education expenditure, and transportation infrastructure, all with q-values surpassing 0.12. Within the central region, q-values of investment in information infrastructure and transportation infrastructure all exceed 0.28. In the western region, all six driving factors notably impact digital rural development. Particularly, q-values for investment in information infrastructure and fiscal expenditure on education surpass 0.40. We also found that all the interactions among driving factors can amplify their influence on the level of DVC. When the driving factors such as investment in information infrastructure, fiscal expenditure on education, and transportation infrastructure interact with other driving factors, significant multiplier effects can be generated.

### Policy recommendations

Based on the aforementioned findings, the following policy suggestions are proposed:

First, interregional policies for DVC need to be established. As previously stated, one of the main structural characteristics of China’s DVC network is that the eastern region occupies a central position, with a relatively high level of digital village development, while the western, central regions have lower scores in terms of outdegree, outcloseness centrality, and middle centrality. That is to say, the level of DVC in most central and western provinces remains comparatively modest. To further optimize the spatial network of China’s DVC and narrow the development gap between regions, establishing a comprehensive, cross-regional mechanism for interregional collaboration in DVC is crucial. Reducing regional disparities is an inevitable requirement for promoting the overall digital rural construction in China. Eastern provinces initiated digital rural construction earlier, garnering substantial experience and technology. Consequently, it is imperative to create channels for interaction in funds, technology, and other resources among eastern and other regions, and to reduce imbalances in DVC development resources across regions. Furthermore, talent scarcity in DVC hampers the development of digital villages in the central and western regions. Implementing talent exchange channels among regions could mitigate this challenge by utilizing expertise from the eastern regions to bolster DVC in the other regions. Establishing an interregional digital agricultural distribution network is also necessary, as it enhances the efficiency of agricultural product circulation among regions, thereby increasing the overall income levels of rural residents.

Second, formulating customized development strategies for DVC in various regions is crucial. Although the eastern coastal regions have relatively well-developed infrastructure for digital rural development, with clear technological and financial advantages, the overall level of digital village development is not yet ideal. Hence, eastern coastal provinces and municipalities should continue to address their shortcomings in DVC by enhancing the digitalization of agricultural tools and methods, as well as the digitalization of the agricultural product distribution networks. Currently, many rural areas in the central and western regions are with relatively poor digital infrastructure, especially in remote mountainous areas. Enhancing investment in the digital infrastructure in these areas is of significance. In particular, accelerating the digital upgrade of rural infrastructure such as irrigation, roads, and electricity is advisable. Without a solid digital infrastructure, many digital technologies cannot be widely applied. In the DVC spatial association network, the eastern region primarily acts as an agent block and main outflow block. Hence it should leverage its technological, informational, and financial advantages to form radiating effects on the DVC in other regions. The central and western regions primarily assume the roles of main inflow and bidirectional spillover. Therefore, these regions should proactively integrate into the national spatial correlation network of digital village development, and engage in the exchange of funds, technology, talent, and energy with the eastern regions.

Third, the distinct driving effects of different driving factors, as well as the amplification effects of factor interaction, should be paid attention. Local governments should prioritize key driving factors when investing in DVC. Based on the findings of Geodetector analysis, the eastern region can prioritize enhancing education, nurturing scientific and technological advancements, stimulating economic growth, and directing additional investments toward transportation infrastructure. The central region should focus its attention on pivotal driving factors like the level of scientific and educational advancement, investment in information infrastructure, and transportation infrastructure. At the same time, the western region should give precedence to driving factors such as investment in information infrastructure, fiscal allocations for education, and transportation networks. Additionally, the phenomenon where interactions among driving factors often magnify their impact on the level of DVC should not be overlooked. Although level of social consumption and level of economic development do not contribute ideally to DVC in China, their explanatory power increases when interacting with other variables. For instance, the q-values of scientific and educational development and level of economic development are 0.0089 and 0.0038 respectively. The interaction effect of these two driving factors (X2∩X3 = 0.109) is stronger than the effect of either factor alone. Hence, as local governments execute investment strategies for digital rural development, they can distribute resources across various factors simultaneously to leverage the enhancing impact of interactions among diverse driving factors.

## Supporting information

S1 Data (XLSX)

## References

[1] Yang Y, Bao W, Wang Y, et al. Measurement of urban-rural integration level and its spatial differentiation in China in the new century. Habitat International. 2021; 117: 102420. doi: 10.1016/j.habitatint.2021.102420

[2] Zhou J. Employment effects of the Internet on rural residents in the context of the digital village. Academic Journal of Management and Social Sciences. 2023; 3(2): 119–126. doi: 10.54097/ajmss.v3i2.10375

[3] Ministry of Agriculture and Rural Affairs: National Evaluation Report on the Development Level of Digital Agriculture and Rural Areas in Counties. Available online: http://www.moa.gov.cn/xw/zwdt/202011/t20201128_6357206.htm (accessed on 5, January, 2024).

[4] Venkatesh V, Sykes T A. Digital divide initiative success in developing countries: A longitudinal field study in a village in India. Information Systems Research. 2013; 24(2): 239–260. doi: 10.1287/isre.1110.0409

[5] Sutriadi R. Defining smart city, smart region, smart village, and technopolis as an innovative concept in Indonesia’s urban and regional development themes to reach sustainability. Conference Series: Earth and Environmental Science. IOP Publishing. 2018; 202: 012047.

[6] Yiwu Zeng, Yixiang Song, Xiazhen Lin, et al. Discussion on several issues of China’s digital rural construction. Chinese Rural Economy. 2021;(04): 21–35.

[7] Haodong Yin, Peng Huo, and Sangui Wang. Agricultural and rural digital transformation: realistic representation, impact mechanism and promotion strategy. Reform. 2020; 1(12): 48–56.

[8] Sheng Wang, Na Yu, Rui Fu. Digital Rural Construction: Mechanisms, Real Challenges, and Implementation Strategies. Reform. 2021; (04): 45–59.

[9] Jiajia Zhao, Juan Wei, Tianjun Liu. Research on the impact and mechanism of digital rural development on farmers’ entrepreneurship. Chinese Rural Economy. 2023; 1(5): 61–80.

[10] Goyal A. Information, direct access to farmers, and rural market performance in central India. American Economic Journal: Applied Economics. 2010; 2(3): 22–45. doi: 10.1257/app.2.3.22

[11] Pengfei Zhou, Meihong Li. Empowering agricultural economic resilience through digital rural construction: impact mechanisms and empirical investigation. Research World. 2023; 1(9): 15–24.

[12] Construction Fang Y. and analysis of digital village evaluation index system. Shanxi Agricultural Economy. 2020; 275, 21–23.

[13] Shen Y, Wu J, Li D. Research on micro measurement model of digital village based on entropy weight method. Journal of Agricultural Library and Information Science. 2020; 34: 68–76.

[14] Cui K, Feng X. Research on the indicator system design for rural digital economy from the perspective of digital village construction. Research in Agricultural Modernization. 2020; 41: 899–909. doi: 10.13872/j.1000-0275.2020.0079

[15] Zhang H, Du K, Jing B. Research on evaluation of digital rural development readiness under rural revitalization strategy. Journal of Xi’an University of Finance and Economics. 2020; 33, 51–60.

[16] Nigam R, Tripathi G, Priya T, et al. Did Covid-19 lockdown positively affect the urban environment and UN-Sustainable Development Goals? PLoS One. 2022; 17(9): e0274621. doi: 10.1371/journal.pone.0274621 36149918 PMC9506620

[17] Bruederle A, Hodler R. Nighttime lights as a proxy for human development at the local level. PloS one. 2018; 13(9): e0202231. doi: 10.1371/journal.pone.0202231 30183707 PMC6124706

[18] Mahad N F, Che Mat Zain C S Z, Mohd Syahidan S N, et al. The application of Intuitionistic Fuzzy Analytic Hierarchy Process (IFAHP) in solving personnel selection problem. Mathematics in Applied Research. 2022; 3: 29–32.

[19] Wang Y, Xu Z. Evaluation of the human settlement in Lhasa with intuitionistic fuzzy analytic hierarchy process. International Journal of Fuzzy Systems. 2018; 20: 29–44. doi: 10.1007/s40815-017-0422-y

[20] Xu Z, Liao H. Intuitionistic fuzzy analytic hierarchy process. IEEE transactions on fuzzy systems. 2013; 22(4): 749–761. doi: 10.1109/TFUZZ.2013.2272585

[21] Julong D. Introduction to grey system theory. The Journal of Grey System. 1989; 1(1): 1–24.

[22] Shi B, He J, Hu Z., et al. Evaluation of Competitiveness of Listed Securities Firms in China—Based on Time-Series Dynamic Composite Weighting Evaluation Model. Financial Forum. 2018; 23(2): 16.

[23] Zhu C, Su Y, Fan R, et al. Exploring provincial carbon-pollutant emission efficiency in China: An integrated approach with social network analysis and spatial econometrics. Ecological Indicators. 2024; 159: 111662. doi: 10.1016/j.ecolind.2024.111662-111676

[24] Scott J. Social network analysis. Sociology. 1988; 22(1): 109–127.

[25] Haythornthwaite C. Social network analysis: An approach and technique for the study of information exchange. Library & information science research. 1996; 18(4): 323–342. doi: 10.1016/S0740-8188(96)90003-1

[26] Liu P, Qin Y, Luo Y, et al. Structure of low-carbon economy spatial correlation network in urban agglomeration. Journal of Cleaner Production. 2023; 394: 136359–136379. doi: 10.1016/j.jclepro.2023.136359

[27] Wang J F, Zhang T L, Fu B J. A measure of spatial stratified heterogeneity. Ecological indicators. 2016; 67: 250–256. doi: 10.1016/j.ecolind.2016.02.052

[28] Xu L, Du H, Zhang X. Driving forces of carbon dioxide emissions in China’s cities: An empirical analysis based on the geodetector method. Journal of Cleaner Production. 2021; 287: 125169. doi: 10.1016/j.jclepro.2020.125169

[29] Shen F. The Endogenous development model of digital village: practical logic, operation mechanism and optimization strategy. E-Government. 2021; 226: 57–67.

[30] Leong C, Pan S, Newell S, Cui L. The Emergence of self-organizing e-commerce ecosystems in remote villages of China:a tale of digital empowerment for rural development. Mis Quarterly. 2016; 40(2): 475–484.

[31] Guo H, Chen X. Building a digital village to promote rural revitalization. Hangzhou Wkly. 2018; 47, 10–11.

[32] Wang W, Cheng H, Zhang L. Poverty assessment using DMSP/OLS night-time light satellite imagery at a provincial scale in China[J]. Advances in Space Research. 2012; 49(8): 1253–1264. doi: 10.1016/j.asr.2012.01.025

[33] Yu J, Meng Y, Zhou S, et al. Research on spatial delineation method of urban-rural fringe combining poi and nighttime light data—taking Wuhan city as an example[J]. International Journal of Environmental Research and Public Health. 2023; 20(5): 4395. doi: 10.3390/ijerph20054395 36901406 PMC10001591

[34] PBruederle A, Hodler R. Nighttime lights as a proxy for human development at the local level. PloS one. 2018; 13(9): e0202231. doi: 10.1371/journal.pone.0202231 30183707 PMC6124706

[35] Jing X, Yan Y Z, Yan L, et al. 2017. A novel method for saturation effect calibration of DMSP/OLS stable light product based on GDP grid data in China mainland at city level. Geography and Geo-Information Science, 33(1): 35–3.

[36] Tan S, Hu B, Kuang B, et al. Regional differences and dynamic evolution of urban land green use efficiency within the Yangtze River Delta, China. Land Use Policy. 2021; 106: 105449. doi: 10.3389/fenvs.2022.1098924

[37] He Y Y, Wei Z X, Liu G Q, et al. Spatial network analysis of carbon emissions from the electricity sector in China. Journal of Cleaner Production. 2020; 262: 121193. doi: 10.1016/j.jclepro.2020.121193

[38] Atçıl A, Kami G. Studying professional careers as hierarchical networks: a case study on the careers of chief judges in the Ottoman Empire (1516–1622). Journal of Historical Network Research, 2022; 7(1): 1–32. doi: 10.25517/jhnr.v7i1.113

[39] Li X, Singh Chandel R B, Xia X. Analysis on regional differences and spatial convergence of digital village development level: theory and evidence from China. Agriculture. 2022; 12(2): 164. doi: 10.3390/agriculture12020164

[40] Zhang C, Li Y, Yang L, et al. Does the development of digital inclusive finance promote the construction of digital villages?—an empirical study based on the Chinese experience. Agriculture. 2023; 13(8): 1616. doi: 10.3390/agriculture13081616

[41] Zhang C, Zhang Y, Li Y, et al. Coupling coordination between fintech and digital villages: mechanism, spatiotemporal evolution and driving factors—An empirical study based on China. Sustainability. 2023; 15(10): 8265. doi: 10.3390/su15108265

[42] Zhu L, Meng J, Zhu L. Applying Geodetector to disentangle the contributions of natural and anthropogenic factors to NDVI variations in the middle reaches of the Heihe River Basin. Ecological Indicators. 2020; 117: 106545. doi: %3Cunderline%3Ehttps%3A//doi.org/10.1016/j.ecolind.2020.106545%3C/underline%3E

